# The role of viral co-infections in the severity of acute respiratory infections among children infected with respiratory syncytial virus (RSV): A systematic review and meta-analysis

**DOI:** 10.7189/jogh.10.010426

**Published:** 2020-06

**Authors:** You Li, Pallavi Pillai, Fuyu Miyake, Harish Nair

**Affiliations:** Centre for Global Health, Usher Institute, University of Edinburgh, Edinburgh, UK

## Abstract

**Background:**

Respiratory syncytial virus (RSV) is the predominant viral cause of childhood pneumonia. Little is known about the role of viral-coinfections in the clinical severity in children infected with RSV.

**Methods:**

We conducted a systematic literature review of publications comparing the clinical severity between RSV mono-infection and RSV co-infection with other viruses in children under five years (<5y). Clinical severity was measured using the following six clinical outcomes: hospitalisation, length of hospital stay, use of supplemental oxygen, intensive care unit (ICU) admission, mechanical ventilation and deaths. We summarised the findings by clinical outcome and conducted random-effect meta-analyses, where applicable, to quantitatively synthesize the association between RSV mono-infection/RSV co-infection and the clinical severity.

**Results:**

Overall, no differences in the clinical severity were found between RSV mono-infection and RSV co-infection with any viruses, except for the RSV-human metapneumovirus (hMPV) co-infection. RSV-hMPV coinfection was found to be associated with a higher risk of ICU admission (odds ratio (OR) = 7.2, 95% confidence interval (CI) = 2.1-25.1; OR after removal of the most influential study was 3.7, 95% CI = 1.1-12.3). We also observed a trend from three studies that RSV-hMPV coinfections were likely to be associated with longer hospital stay.

**Conclusion:**

Our findings suggest that RSV-hMPV coinfections might be associated with increased risk for ICU admission in children <5y compared with RSV mono-infection but such association does not imply causation. Our findings do not support the association between RSV coinfections with other viruses and clinical severity but further large-scale investigations are needed to confirm the findings.

**Protocol registration:**

PROSPERO CRD42019154761.

Respiratory syncytial virus (RSV) is the predominant viral cause of childhood pneumonia [1-3]. It is estimated that in the year of 2015, there were 33.1 million episodes of RSV-associated acute lower respiratory infections (ALRI) and 3.2 million RSV-ALRI hospital admissions in children under five years of age (<5y) globally [4]. RSV activity is found to be seasonal in most locales globally and associated with low temperature and/or high relative humidity [5]. Individual-level risk factors for RSV-ALRI include prematurity, low birth weight, being male, having siblings, maternal smoking, history of atopy, no breastfeeding and crowding [6].

As new diagnostic techniques for respiratory pathogens became more widely available in research and clinical settings, co-detection of RSV with another respiratory virus is not uncommon. Studies have shown that co-detection of RSV with other respiratory viruses could account for 35%-40% of all RSV infections in young children [7-9]. However, the role of viral-coinfections remains unclear in the severity of RSV-associated respiratory diseases. Several systematic reviews suggest that there is no association between disease severity and viral co-infections (compared to mono-infections) but little was reported in these reviews regarding RSV-specific effects [10-13]. Therefore, we conducted a systematic review and meta-analysis to understand the role of viral-coinfections with RSV in the clinical severity in children <5y.

## METHODS

### Literature search

This systematic review was conducted and reported according to the Preferred Reporting Items for Systematic Reviews and Meta-Analyses (PRISMA) guidelines (checklist in Appendix S1 in the **Online Supplementary Document**) [14]. The protocol of this review was registered in International Prospective Register of Systematic Review (PROSEPRO) with registration number CRD42019154761. We searched three databases, Medline (Ovid), Embase (Ovid) and Global Health (Ovid) for publications by 31st December 2019 (with no limits on the start year) that reported morbidity and mortality by RSV-specific mono-infection and co-infection in children <5y. Additional references from citation searching were also considered for inclusion.

We considered the following outcomes a priori in our review: being hospitalised, length of hospital stay, use of supplemental oxygen, intensive care unit (ICU) admission, mechanical ventilation and deaths. The search strategy (Appendix S2 in the **Online Supplementary Document**) is featured by the combination of terms including RSV, coinfection/mixed infection/superinfection, and the outcomes as stated above. We did not include age-specific search terms in the search strategy as studies including a wider age group (eg, all ages) could still have reported sub-age-groups that are eligible to be included in our review. No restrictions were applied to the language of the publications.

### Selection criteria

#### Inclusion criteria

- Population-based studies reporting any laboratory-confirmed viral respiratory coinfection with RSV and mono respiratory infection of RSV in children <5y or any sub-age-groups; AND
- At least one of the following outcomes should be reported separately in coinfection group and mono-infection group: being hospitalised, length of hospital stay, use of supplemental oxygen, ICU admission, mechanical ventilation and deaths.

#### Exclusion criteria

- Studies that only reported nosocomial infections; OR
- Studies that only included children with comorbidities or preterm children; OR
- Studies that reported fewer than 10 cases; OR
- Reviews or studies reporting data that were previously reported by another study.

### Literature selection and extraction

Based on the selection criteria above, two reviewers (YL and PP) independently screened titles, abstracts and full-texts of the retrieved records from the literature search. For data extraction, a questionnaire was tailored to collect relevant information and results from included studies. The questionnaire consists of two forms, data cataloguing and data extraction. Data cataloguing form collects general information of the study design, study subjects, diagnostic test and statistical method. Data extraction form collects results of comparisons in the outcomes between mono-infection group and coinfection group. More details on the data cataloguing and extraction forms are available in Appendix S3 in the **Online Supplementary Document**. Two reviewers (PP and FM) independently conducted the data extraction. Any inconsistencies were resolved among YL, PP and FM.

### Quality assessment

Quality assessment was conducted for all included studies independently by two reviewers (PP and FM). The questionnaire used for the quality assessment was modified based on the Critical Appraisal Skills Programme (CASP) checklist for cohort studies. The questionnaire contained the following seven questions: 1. Did the study address a clearly focused issue?, 2. Were the subjects recruited in an acceptable way?, 3. Was the exposure accurately measured to minimise bias?, 4. Was the outcome accurately measured to minimise bias?, 5. Have the authors taken account of any confounding factors in the design and/or analysis?, 6. Can the results be applied to the local population?, 7. Do the results of this study fit with other available evidence? Answer to each of the question could be “Yes”, “No”, or “Can’t tell”.

### Data analysis

For each outcome, a narrative summary of results was conducted comparing mono-infection group and co-infection group. The summary was further stratified by pathogen pair if two or more studies were available for a specific pathogen pair. For the outcome length of hospital stay, no meta-analysis was conducted due to the reporting variability (eg, median and range, median and interquartile range, mean and standard deviation, etc.). For outcomes other than length of hospital stay, crude odds ratios (OR) were either extracted or calculated from the original results; a random-effect meta-analysis of ORs was conducted if three or more studies were available per co-infected virus and outcome. The choice of conducting a random-effect meta-analysis (rather than fixed-effect meta-analysis) was based on the anticipation that populations included in the studies differed by ethnicity, age, clinical diagnosis and hospital setting (eg, outpatient, emergency department, inpatient, etc.). Ad hoc sensitivity analysis was conducted for statistically significant meta-analysis results by taking out one study each time from the analysis (ie, the leave-one-out method). Publication bias was assessed by visual inspection of the funnel plot and was tested by Egger’s regression method [15]. All statistical analyses were conducted using the R software (version 3.5.2) [16].

## RESULTS

**Figure 1:**
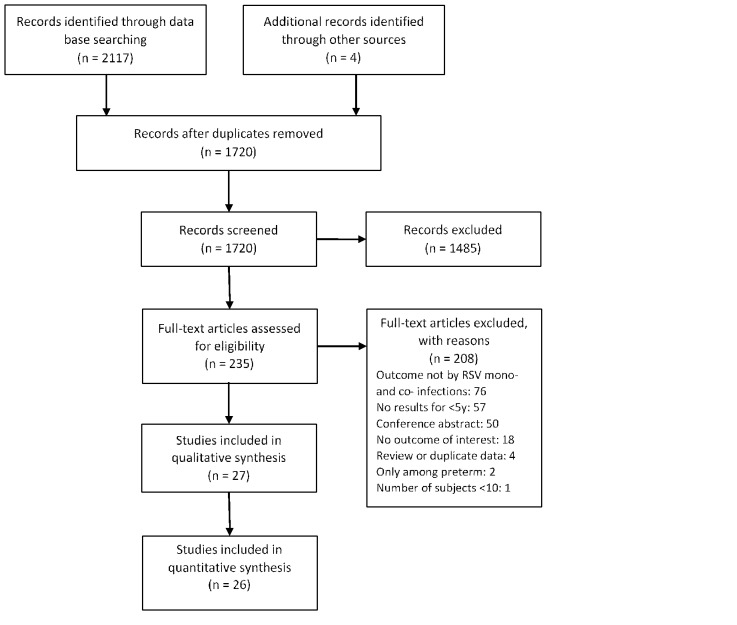
PRISMA diagram for selection of studies.

After removal of duplicates, we screened 1720 studies by title and abstract and screened 235 studies by full-text for eligibility. The percentage of agreement between the two reviewers during the full-text screening stage was 93% (218/235). A total of 27 studies [7,8,17-41] were included in our review (**Figure 1**). General information and quality assessment of the included studies is available in Appendix S4 in the **Online Supplementary Document**. No studies were excluded based on the quality assessment. PCR was used to detect RSV and other viruses in all of the 27 studies. Heterogeneity was observed among populations in the studies in terms of country, age group, clinical diagnosis, and setting (eg, outpatient, inpatient, etc.). Eight studies [8,23,27-30,32,38] used statistical models to account for common confounders such as age, sex, prematurity and comorbidity. Among studies reporting both unadjusted and adjusted results, no substantial differences were observed between the unadjusted and adjusted results (Table S1 in the **Online Supplementary Document**) [8,23,30].

### Hospitalisation

Three studies [29,31,36] compared the proportion of being hospitalised between RSV mono-infection and co-infection. Their findings, however, were not consistent with one study supporting increased hospitalisation in co-infection group (OR = 2.67, 95% CI = 1.71-4.18) [31], one study supporting decreased hospitalisation (OR = 0.42, 95% CI = 0.30-0.59) [36], and another study supporting no difference [29]. (Table S2 in the **Online Supplementary Document**).

### Length of hospital stay

Seventeen studies [7,18-24,26-28,32,33,37,38,40,41] reported the length of hospital stay in the mono- and co-infection groups. Although these studies reported the results in various statistical measures, most of the results were in support for no association between co-infections and length of hospital stay. There was a trend that co-infections with human metapnuemovirus (hMPV) were likely to be associated with longer hospital stay but not yet statistically significant. (**Table 1**)

**Table 1:** Summary of results on the length of hospital stay (in days), grouped by co-infected virus

Study	Virus coinfected	Mono-infection	Co-infection	Notes
**De Paulis, 2011 [****23****]**	Any viruses	8 (6-10)	8 (6-12)	Median (IQR)
**Espinola, 2012 [****18****]**	Any viruses	2-12	2-15	Range
**Falkenstein-Hagander, 2014 [****41****]**	Any viruses	5.0 (3.0-6.0)	3.0 (3.0-5.0)	Median (IQR)
**Gagliardi, 2013 [****19****]**	Any viruses	8	8	Median
**Gokce, 2018 [****26****]**	Any viruses	7.2 ± 3.8	7.5 ± 4.7	Mean±SD
**Janahi, 2017 [****27****]**	Any viruses	81%	88%	Proportion of ≥4 d of stay
**Kelly, 2015 [****28****]**	Any viruses	5.1(2.1-8.0)	4.0(2.1-9.0)	Median (IQR)
**Mansbach, 2012 [****20****]**	Any viruses	48%	51%	Proportion of ≥3 d of stay
**Petrarca, 2018 [****33****]**	Any viruses	5 (1-27)	5 (2-12)	Median (range)
**Yu, 2010 [****37****]**	Any viruses	7.2 ± 3.6	7.2 ± 2.0	Mean±SD
**Aberle, 2005 [****21****]**	RV	7.6 ± 0.3	7.4 ± 0.4	Mean±SEM
**de Silva, 2013 [****7****]**	RV	6.1* in 0-5m, 5.9* in 6m-3y	10.8 ± 3.4 in 0-5m, 5.6 ± 0.5 in 6m-3y	Mean±SD
**Janahi, 2017 [****27****]**	RV	81%	84%	Proportion of ≥4 d of stay
**Kwon, 2019 [****38****]**	RV	5 (2-12)	4 (3-13)	Median (range)
**Mansbach, 2012 [****20****]**	RV	48%	54%	Proportion of ≥3 d of stay
**Marguet, 2009 [****32****]**	RV	6 (5-8)	5 (4-6)	Median (IQR)
**Petrarca, 2018 [****33****]**	RV	5 (1-27)	5 (3-9)	Median (range)
**Janahi, 2017 [****27****]**	Any viruses minus RV	81%	91%	Proportion of ≥4 d of stay
**Mansbach, 2012 [****20****]**	Any viruses minus RV	48%	47%	Proportion of ≥3 d of stay
**Ali, 2010 [****22****]**	hMPV	4 (3-6.2)	8.5 (4-9.7)	Median (IQR)
**Caracciolo, 2008 [****40****]**	hMPV	3.5 ± 3.0	4.1 ± 2.1	Mean±SD
**Foulongne, 2006 [****24****]**	hMPV	4 (2-5)	7 (4-8)	Median (IQR)
**Aberle, 2005 [****21****]**	ADV	7.6 ± 0.3	7.8 ± 0.9	Mean±SEM

RV – rhinovirus, hMPV – human metapneumovirus, ADV – adenovirus, IQR – interquartile range, SD – standard deviation, SEM – standard error of the mean, m – month, y – year

*Standard deviation was not available.

### Use of supplemental oxygen

**Figure 2:**
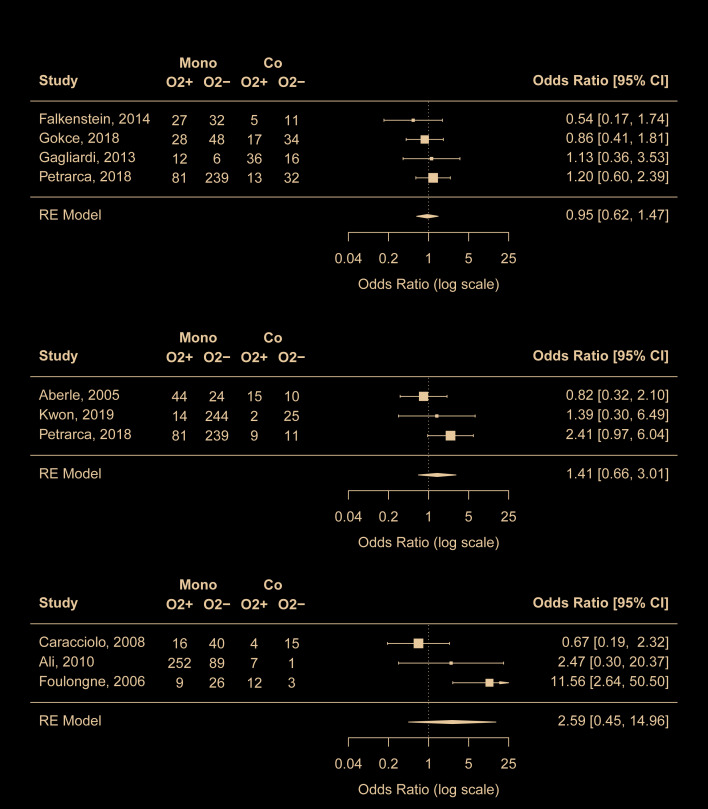
Comparison of risk for use of supplement oxygen between RSV mono-infection and A) RSV coinfections with any viruses, B) RSV coinfections with rhinovirus, C) RSV coinfections with human metapneumovirus. Mono – mono-infection, Co – co-infection, O2 – use of supplement oxygen.

Nine studies [19,21,22,24,26,33,38,40,41] reported the use of supplemental oxygen in mono- and co-infection groups. According to the meta-analyses results, RSV coinfections with any viruses, with rhinovirus, or with hMPV did not seem to be associated with increased use of supplemental oxygen compared with RSV mono-infection (**Figure 2**). No risk of publication bias was identified. Outside the meta-analyses, one study [21] reported that RSV coinfections with adenovirus was associated with decreased use of supplemental oxygen (OR = 0.44, 95% CI = 0.25-0.77).

### ICU admission

**Figure 3:**
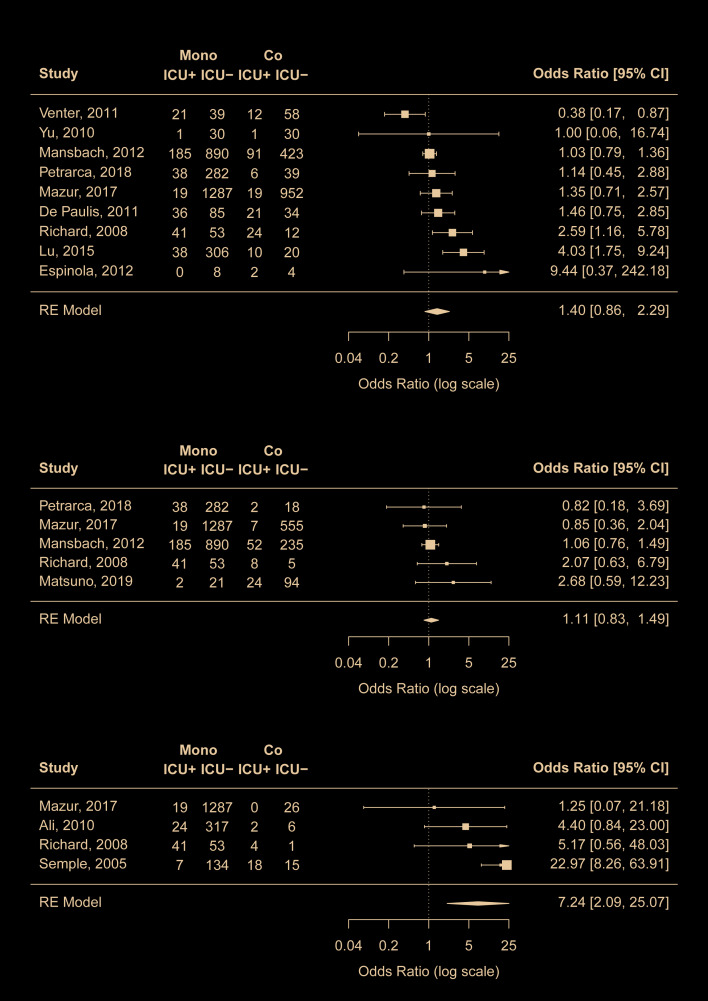
Comparison of risk for ICU admission between RSV mono-infection and A) RSV coinfections with any viruses, B) RSV coinfections with rhinovirus, C) RSV coinfections with human metapneumovirus. Mono – mono-infection, Co – co-infection, ICU – intensive care unit.

Twelve studies [8,18,20,22,23,30,33-37,39] compared the proportion of ICU admission between mono- and co-infection groups. While RSV coinfections with any viruses or with rhinovirus did not seem to be associated with ICU admission, coinfections with hMPV were found to be highly associated with increased risk of ICU admission compared to admission to regular wards (pooled OR = 7.2, 95% CI = 2.1-25.1) (**Figure 3**). To further validate the findings on hMPV-RSV coinfection, we conducted leave-one-out ad hoc sensitivity analyses and found that the pooled OR remained statistically significant even after the removal of the study by Semple et al [35] that appeared to be the most important driver of the results (OR = 3.7, 95% CI = 1.1-12.3). No risk of publication bias was identified.

Outside the meta-analysis above, RSV coinfections with adenovirus (two studies [8,34]), with human coronavirus-NL63 (one study [34]), and with parainfluenza virus (two studies [8,34]) were found to be associated with higher risk for ICU admission (Table S3 in the **Online Supplementary Document**).

### Mechanical ventilation

**Figure 4:**
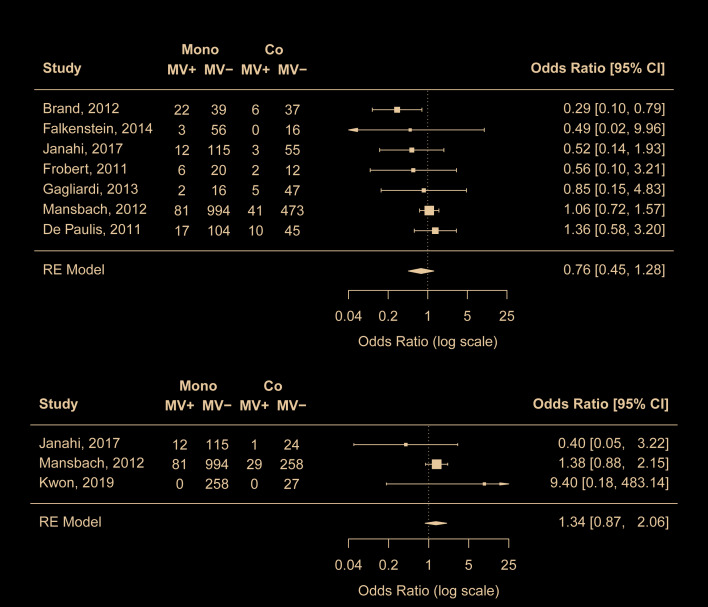
Comparison of risk for mechanical ventilation between RSV mono-infection and A) RSV coinfections with any viruses, B) RSV coinfections with rhinovirus. Mono – mono-infection; Co – co-infection; MV – mechanical ventilation.

Nine studies [17,19,20,22,23,25,27,38,41] compared the proportion of mechanical ventilation in mono- and co-infection groups. Meta-analyses results suggest that RSV coinfections with any viruses or with rhinovirus was not associated with increased risk of mechanical ventilation (**Figure 4**). No risk of publication bias was identified.

### Deaths

Four studies [22,28,36,39] reported deaths in mono- and co-infection groups. These studies reported very small number of deaths (range: 3-8) and the results were mixed. (Table S4 in the **Online Supplementary Document**).

## DISCUSSION

To our knowledge, this is the first systematic review and meta-analysis focusing on the role of viral co-infections in the clinical severity among RSV infected young children. Our findings suggest no association between viral co-infections with RSV and clinical severity, except for RSV and hMPV co-infections, in which this specific pair is found to be associated with higher risk of ICU admission.

Similar to the findings of previous reviews on non-RSV-specific viral-coinfections [10-13], we found no differences in the clinical severity between young children with RSV mono-infection and RSV co-infections with any viruses combined. Nonetheless, different virus pairs could have different types and subtypes of interactions [42]; as a result, their effects on clinical severity could differ and were masked from non-virus-specific results. To this end, we conducted separate analyses and summary by virus pair where applicable. We found that young children with RSV and hMPV coinfection were more likely to be admitted to ICU than those with RSV mono-infection in both the main analysis and the ad hoc leave-one-out analysis. We also observed a trend from three studies [21,24,40] that RSV and hMPV coinfections were likely to be associated with longer hospital stay. The observed increase in clinical severity of RSV-hMPV coinfection was unlikely due to the pathogenic effect of hMPV alone as no statistical significant difference was observed in the clinical severity of RSV mono-infection and hMPV mono-infection [22,24,43]. These findings could have important implications for the clinical management of children who were tested positive for both viruses. However, there are several caveats about the observed association between RSV-hMPV coinfection and clinical severity. First of all, the observed association alone does not indicate causation. Second, there is still a lack of biological explanation for the pathogenic effect of this specific virus pair. Third, increased clinical severity in RSV-hMPV coinfection was not observed for all of the outcomes in our review. For example, RSV-hMPV coinfection was not found to be associated with increased use of supplement oxygen; this could be due to either lack of power (ie, false negative) or no association.

We found that common confounders of the respiratory virus and disease association, such as age, sex, prematurity and comorbidity, did not seem to substantially affect the findings of the association between RSV-coinfections and the clinical severity [8,23,30]. Nonetheless, confounding effect was observed in these studies with a more than 10% of change in the estimates, with two studies [23,30] showing positive confounding and one showing negative confounding [8]. In addition to the aforementioned confounders, bacterial co-infection could also confound the observed effect but this was not reported in any of the included studies [44]. For example, pneumococcal infection was found to be associated with RSV infection and with increased clinical severity [45,46].

Our study is not without limitations. First, although our results did not support the association between other virus pairs with RSV and clinical severity in young children, we lacked the power to reach a firm conclusion of no association. The numbers of RSV-coinfections in most studies were less than 50. More large-scale studies are warranted to confirm our findings. Second, we were unable to differentiate co-detection from co-infection. Co-detection of RSV with the other virus does not necessarily implicate pathogenic effect of either virus. Detection of RSV in the respiratory tract was found to be highly associated with ALRI in children whereas other viruses, such as adenovirus and rhinovirus, when presented alone, were less associated with ALRI and were more often detected in healthy children [47]. This might partly explain our findings on RSV co-infections with rhinovirus and with any viruses. Third, due to limited data from the literature, we were unable to conduct subgroup analysis by comorbidity status or by prematurity that might act as a modifier of the clinical severity. Fourth, for random-effect meta-analysis, estimation for between-study heterogeneity is likely to be biased towards zero when the number of studies is very small (eg, three) [48]; as a result, the pooled estimate is more sensitive to outliers.

Despite these limitations, our study is the first systematic review that compares the effects of RSV-specific coinfection on the clinical severity in children <5y. Our study results suggest that RSV-hMPV coinfections are associated with higher risk of ICU admission and this could have relevance to the clinical management of paediatric patients with RSV-hMPV coinfection. No firm conclusions can be made regarding RSV coinfections with other viruses. Further well-designed studies are necessary to confirm our findings.

## Additional material

Online Supplementary Document
