# Supplementary material for: The role of viral co-infections in the severity of acute respiratory infections among children infected with respiratory syncytial virus (RSV): A systematic review and meta-analysis
Source: J Glob Health. 2020 Jun 11;10(1):010426. doi: 10.7189/jogh.10.010426 (PMC7295447; doi:10.7189/jogh.10.010426)
Supplement: Online Supplementary Document [file jogh-10-010426-s001.pdf]

# Appendix S1. PRISMA Checklist

| Section/topic | # | Checklist item | Reported on page # |
|---|---|---|---|
| **TITLE** | | | |
| Title | 1 | Identify the report as a systematic review, meta-analysis, or both. | 1 |
| **ABSTRACT** | | | |
| Structured summary | 2 | Provide a structured summary including, as applicable: background; objectives; data sources; study eligibility criteria, participants, and interventions; study appraisal and synthesis methods; results; limitations; conclusions and implications of key findings; systematic review registration number. | 1-2 |
| **INTRODUCTION** | | | |
| Rationale | 3 | Describe the rationale for the review in the context of what is already known. | 3 |
| Objectives | 4 | Provide an explicit statement of questions being addressed with reference to participants, interventions, comparisons, outcomes, and study design (PICOS). | 3 |
| **METHODS** | | | |
| Protocol and registration | 5 | Indicate if a review protocol exists, if and where it can be accessed (e.g., Web address), and, if available, provide registration information including registration number. | 3 |
| Eligibility criteria | 6 | Specify study characteristics (e.g., PICOS, length of follow-up) and report characteristics (e.g., years considered, language, publication status) used as criteria for eligibility, giving rationale. | 3-4 |
| Information sources | 7 | Describe all information sources (e.g., databases with dates of coverage, contact with study authors to identify additional studies) in the search and date last searched. | 3 |
| Search | 8 | Present full electronic search strategy for at least one database, including any limits used, such that it could be repeated. | Appendix pp 3-5 |
| Study selection | 9 | State the process for selecting studies (i.e., screening, eligibility, included in systematic review, and, if applicable, included in the meta-analysis). | 4 |
| Data collection process | 10 | Describe method of data extraction from reports (e.g., piloted forms, independently, in duplicate) and any processes for obtaining and confirming data from investigators. | 4 |
| Data items | 11 | List and define all variables for which data were sought (e.g., PICOS, funding sources) and any assumptions and simplifications made. | 4 and Appendix p6 |
| Risk of bias in individual studies | 12 | Describe methods used for assessing risk of bias of individual studies (including specification of whether this was done at the study or outcome level), and how this information is to be used in any data synthesis. | 4-5 |
| Summary measures | 13 | State the principal summary measures (e.g., risk ratio, difference in means). | 5 |
| Synthesis of results | 14 | Describe the methods of handling data and combining results of studies, if done, including measures of consistency (e.g., $I^2$) for each meta-analysis. | 5 |

| Section/topic | # | Checklist item | Reported on page # |
|---|---|---|---|
| Risk of bias across studies | 15 | Specify any assessment of risk of bias that may affect the cumulative evidence (e.g., publication bias, selective reporting within studies). | 5 |
| Additional analyses | 16 | Describe methods of additional analyses (e.g., sensitivity or subgroup analyses, meta-regression), if done, indicating which were pre-specified. | 5 |
| **RESULTS** | | | |
| Study selection | 17 | Give numbers of studies screened, assessed for eligibility, and included in the review, with reasons for exclusions at each stage, ideally with a flow diagram. | 5 |
| Study characteristics | 18 | For each study, present characteristics for which data were extracted (e.g., study size, PICOS, follow-up period) and provide the citations. | 5 and Appendix pp 7-8 |
| Risk of bias within studies | 19 | Present data on risk of bias of each study and, if available, any outcome level assessment (see item 12). | Appendix p9 |
| Results of individual studies | 20 | For all outcomes considered (benefits or harms), present, for each study: (a) simple summary data for each intervention group (b) effect estimates and confidence intervals, ideally with a forest plot. | 5-6 and Appendix p10 |
| Synthesis of results | 21 | Present results of each meta-analysis done, including confidence intervals and measures of consistency. | 14-16 |
| Risk of bias across studies | 22 | Present results of any assessment of risk of bias across studies (see Item 15). | 6 |
| Additional analysis | 23 | Give results of additional analyses, if done (e.g., sensitivity or subgroup analyses, meta-regression [see Item 16]). | 6 |
| **DISCUSSION** | | | |
| Summary of evidence | 24 | Summarize the main findings including the strength of evidence for each main outcome; consider their relevance to key groups (e.g., healthcare providers, users, and policy makers). | 7 |
| Limitations | 25 | Discuss limitations at study and outcome level (e.g., risk of bias), and at review-level (e.g., incomplete retrieval of identified research, reporting bias). | 7 |
| Conclusions | 26 | Provide a general interpretation of the results in the context of other evidence, and implications for future research. | 8 |
| **FUNDING** | | | |
| Funding | 27 | Describe sources of funding for the systematic review and other support (e.g., supply of data); role of funders for the systematic review. | 8 |

*From:* Moher D, Liberati A, Tetzlaff J, Altman DG, The PRISMA Group (2009). Preferred Reporting Items for Systematic Reviews and Meta-Analyses: The PRISMA Statement. PLoS Med 6(7): e1000097. doi:10.1371/journal.pmed1000097

For more information, visit: **www.prisma-statement.org**.

# Appendix S2. Search Strategy

## MEDLINE

1. *respiratory syncytial viruses/ or *respiratory syncytial virus, human/ or RSV.mp. or respiratory syncytial virus*.mp.

2. *Respiratory Syncytial Virus Infections/

3. Influenza, Human/ or influenza.mp. or flu.mp.

4. exp influenzavirus a/ or exp influenzavirus b/ or exp influenzavirus c/

5. 1 or 2 or 3 or 4

6. coinfection*.mp. or exp Coinfection/ or mixed infection*.mp.

7. superinfection*.mp. or exp Superinfection/

8. 6 or 7

9. exp morbidity/ or exp mortality/ or exp death/

10. exp critical care/ or exp hospitalization/

11. *Hypoxia/

12. exp oxygen inhalation therapy/ or exp respiration, artificial/

13. 9 or 10 or 11 or 12

14. 5 and 8 and 13

341 Results by 26th Mar 2019 (first search)

389 Results by 31st Dec 2019

## EMBASE

1. *respiratory syncytial viruses/ or *respiratory syncytial virus, human/ or RSV.mp. or respiratory syncytial virus*.mp.

2. *Respiratory Syncytial Virus Infections/

3. Influenza, Human/ or influenza.mp. or flu.mp.

4. exp influenzavirus a/ or exp influenzavirus b/ or exp influenzavirus c/

5. 1 or 2 or 3 or 4

6. coinfection*.mp. or exp Coinfection/ or mixed infection*.mp.

7. superinfection*.mp. or exp Superinfection/

8. 6 or 7

9. exp morbidity/ or exp mortality/ or exp death/

10. exp critical care/ or exp hospitalization/

11. *Hypoxia/

12. exp oxygen inhalation therapy/ or exp respiration, artificial/

13. 9 or 10 or 11 or 12

14. 5 and 8 and 13

1300 Results by 26th Mar 2019 (first search)

1439 Results by 31st Dec 2019

13. 6 and 10 and 12

# Sample forms used for data cataloguing and extraction

## Sample data cataloguing form

| | |
|---|---|
| *Study ID* | |
| *Author* | |
| *Year of publication* | |
| *Study period: from* | |
| *Study period: to* | |
| *Region* | |
| *Country* | |
| *Setting (community/hospital)* | |
| *Wards (inpatient/outpatient/emergency room)* | |
| *Age* | |
| *Number of subjects* | |
| *Diagnosis or symptoms* | |
| *Specimen for virus detection* | |
| *Diagnostic method for virus detection* | |
| *Hospitalisation (available or not)* | |
| *Length of stay (available or not)* | |
| *ICU admission (available or not)* | |
| *Mechanical ventilation (available or not)* | |
| *Use of supplemental oxygen (available or not)* | |

| | |
|---|---|
| *Mortality (available or not)* | |
| *Other_outcomes_reported (yes or no)* | |
| *Other_outcomes (free texts)* | |
| *Baseline variables compared between mono-infection and co-infection (e.g. age, sex, etc.)* | |
| *Any baseline variables balanced between the two groups* | |

## Appendix S3. Sample data extraction form

| *Questions* | *RSV Mono-infection* | *RSV Coinfection* |
|---|---|---|
| *Study ID* | | |
| *Virus* | | |
| *Number of cases* | | |
| *Results on proportion of hospitalisation* | | |
| *Results on length of stay* | | |
| *Results on ICU admission* | | |
| *Results on mechanical ventilation* | | |
| *Results on use of supplemental oxygen* | | |
| *Results on mortality* | | |
| *Other results reported (free texts)* | | |

## Appendix S4. Characteristics of included studies

| Author | Year of Publication | Study Period | Country | Age | Diagnostic method | Clinical diagnosis | Setting(s) | Hospital admission | Length of hospital stay | Use of supplemental oxygen | ICU admission | Mechanical ventilation | Deaths |
|---|---|---|---|---|---|---|---|---|---|---|---|---|---|
| **Aberle** | 2005 | OCT 2010–JUL 2014 | Austria | <1y | PCR | ALRI | IP | No | Yes | Yes | No | No | No |
| **Ali** | 2010 | JAN 2007–MAR 2007 | Jordan | <5y | PCR | ARI | IP | No | Yes | Yes | Yes | Yes | Yes |
| **Brand** | 2012 | NOV 2006–APR 2009 | Netherlands | <2y | PCR | Bronchiolitis | ER + IP | No | No | No | No | Yes | No |
| **Caracciolo** | 2008 | OCT 2005–APR 2007 | Italy | <5y | PCR | ARI | IP | No | Yes | Yes | No | No | No |
| **da Silva** | 2013 | APR 2007–NOV 2007 | Brazil | <3y | PCR | ALRI | ER + IP | No | Yes | No | No | No | No |
| **De Paulis** | 2011 | FEB 2005–NOV 2005 | Brazil | <2y | PCR | ARI | IP | No | Yes | No | Yes | Yes | No |
| **Espinola** | 2012 | MAY 2010–OCT 2011 | Paraguay | <2y | PCR | ARI | IP | No | Yes | No | Yes | No | No |
| **Falkenstein-Hagander** | 2014 | SEP 2006–MAY 2007 | Sweden | <1y | PCR/IFA | ALRI | IP | No | Yes | Yes | No | Yes | No |
| **Foulongne** | 2006 | NOV 2003–OCT 2004 | France | <5y | PCR/DFA | RTD | IP | No | Yes | Yes | No | No | No |
| **Frobert** | 2011 | DEC 2008–MAR 2009 | France | <2y | PCR | RTD | ICU | No | No | No | No | Yes | No |
| **Gagliardi** | 2013 | JAN 2005–DEC 2005 | Brazil | <5y | PCR/DFA | ARI | IP + OP + ICU | No | Yes | Yes | No | Yes | No |
| **Gokce** | 2018 | JAN 2013–OCT 2016 | Turkey | <2y | PCR | Bronchiolitis | IP | No | Yes | Yes | No | No | No |
| **Janahi** | 2017 | JAN 2010–DEC 2011 | Qatar | 2w–2y | PCR | Bronchiolitis | IP | No | Yes | No | No | Yes | No |
| **Kelly** | 2015 | APR 2012–AUG 2014 | Botswana | <2y | PCR | Pneumonia | IP | No | Yes | No | No | No | Yes |
| **Kwon** | 2019 | OCT 2014–APR 2017 | South Korea | <5y | PCR | ARI | IP | No | Yes | Yes | No | Yes | No |

| | | | | | | | | | | | | | |
|---|---|---|---|---|---|---|---|---|---|---|---|---|---|
| **Lim** | 2017 | JAN 2008 – DEC 2012 | Australia | 6–59m | PCR/ culture | ARI | ER + IP | Yes | No | No | No | No | No |
| **Lu** | 2015 | JAN 2010–DEC 2014 | China | <1m | PCR/DFA | ALRI | IP | No | No | No | Yes | No | No |
| **Macao** | 2011 | NOV 2009–MAR 2009 | Portugal | <2y | PCR/DFA | Bronchiolitis | ER + IP | Yes | No | No | No | No | No |
| **Mansbach** | 2012 | NOV 2007–MAR 2010 | US | <2y | PCR | Bronchiolitis | ER + IP | No | Yes | No | Yes | Yes | No |
| **Marguet** | 2009 | NOV 2002–MAR 2004 | France | 1m–1y | PCR/DFA | Bronchiolitis | ER + IP | No | Yes | No | No | No | No |
| **Matsuno** | 2019 | JUN 2008–MAY 2009 | Brazil | <3y | PCR/DFA/ IFA | ALRI | IP | No | No | No | Yes | No | Yes |
| **Mazur** | 2017 | FEB 2009–DEC 2013 | South Africa | <5y | PCR | SARI | IP + ICU | No | No | No | Yes* | No | No |
| **Petrarca** | 2018 | OCT 2004–MAY 2016 | Italy | <1y | PCR | Bronchiolitis | IP | No | Yes | Yes | Yes | No | No |
| **Richard** | 2008 | SEP 2003–APR 2005 | France | <1y | PCR/ culture | Bronchiolitis | IP + ICU | No | No | No | Yes | No | No |
| **Semple** | 2005 | NOV 2001–MAR 2002 | UK | <2y | EIA/PCR | Bronchiolitis | IP + ICU | No | No | No | Yes | No | No |
| **Venter** | 2011 | JAN 2006–DEC 2007 | South Africa | <5y | PCR/DFA/ EIA | ARI | IP + OP | Yes | No | No | Yes | No | Yes |
| **Yu** | 2010 | Winter 2006–Winter 2008 | China | 1–30m | PCR | Bronchiolitis | IP | No | Yes | No | Yes | No | No |

*The authors used a composite outcome of mechanical ventilation, ICU admission and death.

PCR = polymerase chain reaction; DFA = direct immunofluorescence assay; IFA = indirect immunofluorescence assay; EIA = enzyme-linked immunosorbent assay; ALRI = acute lower respiratory infection; ARI = acute respiratory infection; RTD = respiratory tract disease; SARI = severe acute respiratory infection; ER = emergency room; IP = inpatient; OP = outpatient; ICU = intensive care unit.

## Quality assessment

| Author | Year of Publication | Did the study address a clearly focused issue? | Were the subjects recruited in an acceptable | Was the exposure accurately measured to minimise | Was the outcome accurately measured to minimise | Have the authors taken account of any confounding factors in the design and/or | Can the results be applied to the local population? | Do the results of this study fit with other available evidence? |
|---|---|---|---|---|---|---|---|---|

| | | | way? | bias? | bias? | analysis? | | |
|---|---|---|---|---|---|---|---|---|
| **Aberle** | 2005 | yes | yes | yes | yes | no | can't tell | yes |
| **Ali** | 2010 | yes | yes | yes | yes | no | yes | yes |
| **Brand** | 2012 | yes | yes | yes | yes | no | yes | yes |
| **Caracciolo** | 2008 | yes | yes | yes | yes | no | yes | yes |
| **da Silva** | 2013 | yes | yes | yes | yes | no | can't tell | yes |
| **De Paulis** | 2011 | yes | yes | yes | yes | yes | yes | yes |
| **Espinola** | 2012 | yes | yes | yes | yes | no | yes | yes |
| **Falkenstein-Hagander** | 2014 | yes | yes | yes | yes | no | yes | yes |
| **Foulongne** | 2006 | yes | yes | yes | yes | no | yes | yes |
| **Frobert** | 2011 | yes | yes | yes | yes | no | yes | yes |
| **Gagliardi** | 2013 | yes | yes | yes | yes | no | yes | yes |
| **Gokce** | 2018 | yes | yes | yes | yes | no | yes | yes |
| **Janahi** | 2017 | yes | yes | yes | yes | yes | yes | yes |
| **Kelly** | 2015 | yes | yes | yes | yes | yes | yes | yes |
| **Kwon** | 2019 | yes | yes | yes | yes | yes | yes | yes |
| **Lim** | 2017 | yes | yes | yes | yes | yes | yes | yes |
| **Lu** | 2015 | yes | yes | yes | yes | yes | yes | yes |
| **Macao** | 2011 | yes | yes | yes | yes | no | yes | yes |
| **Mansbach** | 2012 | yes | yes | yes | yes | no | yes | yes |
| **Marguet** | 2009 | yes | yes | yes | yes | yes | yes | yes |
| **Matsuno** | 2019 | yes | yes | yes | yes | no | yes | yes |
| **Mazur** | 2017 | yes | yes | yes | yes | yes | no | yes |
| **Petrarca** | 2018 | yes | yes | yes | yes | no | yes | yes |
| **Richard** | 2008 | yes | yes | yes | yes | no | yes | yes |
| **Semple** | 2005 | yes | yes | yes | yes | no | yes | yes |
| **Venter** | 2011 | yes | yes | yes | yes | no | yes | yes |
| **Yu** | 2010 | yes | yes | yes | yes | no | yes | yes |

## Global Health

237 results by 26th Mar 2019 (first search)

289 results by 31st Dec 2019

## Table S1. Summary of studies reporting both unadjusted and adjusted results

| Study | Virus co-infected, Outcome | Unadjusted OR | Adjusted OR |
|---|---|---|---|
| **De Paulis, 2011** | Any viruses,<br>mechanical ventilation | 1.36<br>(95% CI: 0.90–2.05) | 1.05<br>(95% CI: 0.29–3.85) |
| **Lu, 2015** | Any viruses,<br>ICU admission | 4.03<br>(95% CI: 1.76–9.24) | 2.72<br>(95% CI: 1.05–7.07) |
| **Mazur, 2017** | Adenovirus,<br>ICU admission | 2.4<br>(95% CI: 1.2–4.8) | 3.4<br>(95% CI: 1.6–7.2) |

OR=odds ratio; CI=confidence interval; ICU=intensive care unit

## Table S2. Summary of results on hospitalisations

| Study | Virus co-infected | Results | Notes |
|---|---|---|---|
| **Lim, 2017** | Influenza virus | Probability of hospitalisation<br>Mono: 43% (95% CI: 36–51)<br>Co: 55% (95% CI: 35–73) | Marginal model fit from multivariable logistic regression model |
| **Macao, 2011** | Bocavirus | OR: 2.67 (95% CI: 1.71–4.18) | — |
| **Venter, 2011** | Any viruses | OR: 0.42 (95% CI: 0.30–0.59) | — |

CI=confidence interval; Mono=mono-infection; Co=co-infection; OR=odds ratio

## Table S3. Additional results on ICU admission

| Study | Virus co-infected | Results | |
|---|---|---|---|
| **Mazur, 2017** | Adenovirus | Mono: 19/1306 (1.5%)<br>Co: 11/340 (3.2%) | OR: 2.3 (95% CI: 1.6–3.3) |

| | | | |
|---|---|---|---|
| **Richard, 2008** | Adenovirus | Mono: 41/94 (43.6%)<br>Co: 1/1 (100%) | — |
| **Mazur, 2017** | Enterovirus | Mono: 19/1306 (1.5%)<br>Co: 3/186 (3.2%) | OR: 1.1 (95% CI: 0.6–2.1) |
| **Richard, 2008** | Enterovirus | Mono: 41/94 (43.6%)<br>Co: 1/5 (20.0%) | OR: 0.3 (95% CI: 0.1–0.9) |
| **Richard, 2008** | Human coronavirus-NL63 | Mono: 41/94 (43.6%)<br>Co: 4/4 (100%) | — |
| **Mazur, 2017** | Influenza virus | Mono: 19/1306 (1.5%)<br>Co: 0/29 (0%) | — |
| **Richard, 2008** | Influenza virus | Mono: 41/94 (43.6%)<br>Co: 2/4 (50.0%) | OR: 1.3 (95% CI: 0.6–2.7) |
| **Mazur, 2017** | Parainfluenza virus | Mono: 19/1306 (1.5%)<br>Co: 2/43 (4.7%) | OR: 3.3 (95% CI: 1.6–6.9) |
| **Richard, 2008** | Parainfluenza virus | Mono: 41/94 (43.6%)<br>Co: 4/4 (100%) | — |

Mono=mono-infection; Co=co-infection; CI=confidence interval; OR=odds ratio

## Table S4. Summary of results on deaths

| Study | Virus co-infected | Results | |
|---|---|---|---|
| **Ali, 2010** | hMPV | Mono: 3/341 (0.9%)<br>Co: 0/8 (0%) | — |
| **Kelly, 2015** | Any viruses | Mono: 3/89 (3.4%)<br>Co: 0/18 (0%) | — |
| **Matsuno, 2019** | Rhinovirus | Mono: 0/23 (0%)<br>Co: 3/118 (2.5%) | — |

| | | | |
|---|---|---|---|
| **Venter, 2011** | Any viruses | Mono: 6/60 (10.0%)<br>Co: 2/70 (2.9%) | OR: 0.26 (95% CI: 0.11–0.59) |

hMPV=human metapneumovirus; Mono=mono-infection; Co=co-infection; CI=confidence interval; OR=odds ratio
